# Genome Sequences of Clinical Isolates of NDM-1-Producing Klebsiella quasipneumoniae subsp. *similipneumoniae* and KPC-2-Producing Klebsiella quasipneumoniae subsp. *quasipneumoniae* from Brazil

**DOI:** 10.1128/MRA.00089-20

**Published:** 2020-03-05

**Authors:** Bruna Fuga, Louise Cerdeira, Flávio Andrade, Tania Zaccariotto, Fernanda Esposito, Brenda Cardoso, Larissa Rodrigues, Ingrith Neves, Carlos E. Levy, Nilton Lincopan

**Affiliations:** a Department of Microbiology, Institute of Biomedical Sciences, Universidade de São Paulo, São Paulo, Brazil; b One Health Brazilian Resistance Project, São Paulo, Brazil; c Hospital de Clínicas, Universidade Estadual de Campinas, Campinas, Brazil; d Department of Clinical Analysis, School of Pharmaceutical Sciences, Universidade de São Paulo, São Paulo, Brazil; University of Southern California

## Abstract

Klebsiella quasipneumoniae is an emerging pathogen in human medicine. We report draft genome sequences of NDM-1- and KPC-2-producing K. quasipneumoniae strains from inpatients in Brazil. K. quasipneumoniae subsp. *quasipneumoniae* and K. quasipneumoniae subsp. *similipneumoniae* harbored broad resistomes. These data could contribute to a better understanding of acquired resistance in K. quasipneumoniae.

## ANNOUNCEMENT

Klebsiella pneumoniae strains of phylogenetic groups Kp1 to Kp7 have been classified as K. pneumoniae *sensu stricto*, K. quasipneumoniae subsp. *quasipneumoniae*, K. variicola subsp. *variicola*, K. quasipneumoniae subsp. *similipneumoniae*, K. variicola subsp. *tropicalensis*, K. quasivariicola, and K. africanensis, respectively (1). Specifically, K. quasipneumoniae has been recognized as an opportunistic pathogen that can acquire clinically relevant antibiotic resistance genes (2–5). Here, we report draft genome sequences of two Klebsiella quasipneumoniae strains producing KPC-2 and NDM-1 carbapenemases, which confer resistance to all clinically relevant β-lactam antibiotics.

**FIG 1:**
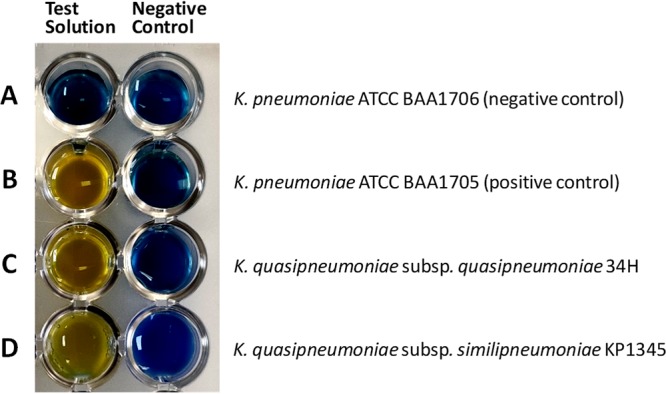
Representative results of the Blue-Carba test for carbapenemase-producing (B, C, and D) and non-carbapenemase-producing (A) bacteria, with test solutions (left) and negative-control solutions (right). (A) K. pneumoniae ATCC BAA1706 (carbapenemase-negative control); (B) K. pneumoniae ATCC BAA1705 (carbapenemase [KPC]-positive control); (C) K. quasipneumoniae subsp. *quasipneumoniae* 34H (this study) (carbapenemase [KPC-2] positive); (D) K. quasipneumoniae subsp. *similipneumoniae* Kp1345 (this study) (carbapenemase [NDM-1] positive). The images were obtained after 2 h of incubation. Carbapenemase production was assessed by the Blue-Carba test method (9), which relies on the detection, in a bacterial extract, of hydrolysis of the carbapenem β-lactam ring through the acidification of a bromothymol blue test solution, used as a color indicator. The test solution consists of an aqueous solution of 0.04% bromothymol blue adjusted to pH 6.0, 0.1 mM ZnSO_4_, and 3 mg/ml imipenem, with a final pH of 7.0. A negative-control solution (0.04% bromothymol blue solution [pH 7.0]) is used to control for the influence of bacterial components or products on the pH of the solution. A loop (approximately 5 μl) of a pure bacterial culture recovered from Mueller-Hinton agar was directly suspended in 100 μl of both test and negative-control solutions in a 96-well microtiter plate and incubated for 2 h at 37°C with agitation (150 rpm). Carbapenemase activity was revealed when the test solution and negative-control wells were yellow and blue, respectively. The non-carbapenemase-producing strain (negative control) remained blue or green with both solutions.

Carbapenem-resistant K. quasipneumoniae strains 34H and Kp1345 were isolated in 2014 from perfusion fluid (6) and in 2017 from a rectal swab for surveillance culture (7), respectively, from patients hospitalized in a teaching hospital in Brazil. Species identification was performed by matrix-assisted laser desorption ionization–time of flight mass spectrometry (8), and antimicrobial susceptibility was determined with the Vitek 2 system (bioMérieux, France) according to the manufacturer’s instructions. Carbapenemase production was detected by the Blue-Carba test (9) (Fig. 1) and modified Hodge test (10), whereas carbapenemase activity of NDM-1 and KPC-2 β-lactamases was confirmed by EDTA and dipicolinic acid inhibition assays, respectively (11–13). Additionally, *bla*_NDM-1_ and *bla*_KPC-2_ genes were identified by PCR amplification and direct DNA sequencing of PCR products (14).

For whole-genome sequencing (WGS) analyses, the strains were streaked to single colonies on MacConkey agar plates and then grown for 18 h at 37°C in 3 ml of lysogeny broth. Total genomic DNA was extracted using a PureLink quick gel extraction kit (Life Technologies, CA) and used for library preparation with a Nextera XT kit (Illumina, San Diego, CA). In addition, the DNA was quantified with a double-stranded DNA high-sensitivity assay using a Qubit 2.0 fluorometer (Life Technologies) according to the manufacturer’s instructions. Subsequently, sequencing was performed on an Illumina NextSeq PE instrument using a paired-end (150-bp) library. The short reads were handled using FastQC v.0.11.3 (http://www.bioinformatics.babraham.ac.uk/projects/fastqc) and Trimmomatic v.0.32 (15). *De novo* assembly was performed using SPAdes v.3.9 (16), and draft genome annotations were made using NCBI PGAP v.3.2 (https://www.ncbi.nlm.nih.gov/genome/annotation_prok). Contamination levels were checked using CheckM v.1.0.3 with default settings (17). WGS data were analyzed using PlasmidFinder v.2.0 (18), ResFinder v.3.2 (19), and SpeciesFinder v.2.0 (20) tools (http://www.genomicepidemiology.org). Default parameters were used for all software.

Genome sequence analysis identified K. quasipneumoniae subsp. *quasipneumoniae* (strain 34H) and K. quasipneumoniae subsp. *similipneumoniae* (strain Kp1345), presenting a total of 16,501,776 and 10,695,728 paired-end reads assembled into 183 and 487 contigs, with 247.0× and 320.0× coverage, respectively. The *N*_50_ values obtained for strains 34H and Kp1345 were 84,397 and 122,604 bp, with GC contents of 57.6% and 56.8%, respectively. In brief, strain 34H presented a genome size calculated as 5,666,228 bp, with 5,134 protein-coding sequences, 82 tRNAs, 22 rRNAs, 12 noncoding RNAs, and 49 pseudogenes, whereas Kp1345 presented a genome size of 5,921,292 bp, with 5,134 protein-coding sequences, 82 tRNAs, 22 rRNAs, 12 noncoding RNAs, and 49 pseudogenes. CheckM results showed 99.99% and 99.938% completeness and 0.952% and 1.061% contamination for the 34H and KPC1345 genomes, respectively.

In summary, we present the draft genome sequences of two carbapenem-resistant Klebsiella quasipneumoniae strains displaying broad resistomes for β-lactams (i.e., *bla*_KPC-2_, *bla*_OKP-A-6_, *bla*_OKP-B-2_, *bla*_NDM-1_, and *bla*_CTX-M-15_) and other medically important antibiotics. These data could contribute to a better understanding of acquired resistance in K. quasipneumoniae.

### Data availability.

The genome sequences of K. quasipneumoniae subsp. *quasipneumoniae* strain 34H and K. quasipneumoniae subsp. *similipneumoniae* strain Kp1345 have been deposited in GenBank under accession numbers NZ_VDFT00000000 (SRA number SRR9950479) and NZ_VDFZ00000000 (SRA number SRR9942580), respectively. For a spreadsheet containing details of antibiotic resistance genes, plasmid incompatibility groups, and CheckM and Qubit results, see Table S1 at https://doi.org/10.6084/m9.figshare.11675805.
